# Metabolic Functional Community Diversity of Associated Bacteria during the Degradation of Phytoplankton from a Drinking Water Reservoir

**DOI:** 10.3390/ijerph17051687

**Published:** 2020-03-05

**Authors:** Sheng-Nan Chen, Pan-Lu Shang, Peng-Liang Kang, Man-Man Du

**Affiliations:** Shaanxi Key Laboratory of Environmental Engineering, Key Laboratory of Northwest Water Resource, Environment and Ecology, MOE, Xi’an University of Architecture and Technology, Xi’an 710055, Shaanxi, China; 18392139665@163.com (P.-L.S.); 18292820381@163.com (P.-L.K.); 18392399725@163.com (M.-M.D.)

**Keywords:** phytoplankton, associated bacteria, BIOLOG, functional diversity

## Abstract

In the drinking water reservoir ecosystem, phytoplankton and bacteria play important roles in shaping freshwater health and function. In this work, the associated bacterial community functional diversity during degradation of phytoplankton was determined using the substrate utilization profiling (BIOLOG) technique, meanwhile, the composition and concentration of phytoplankton were examined using a microscope. The results indicated that *Euglena* decreased 58.33% from 0 to 38 d, while the smallest degradation of *Bacillariophyta* was 20.19%. Average well color development (*AWCD*_590nm_) increased during the static periods from 0 to 38 d; however, the *AWCD*_590nm_ of 18 and 38 d had no significant difference (*p* < 0.05). The Simpson’s index (*D*) was in accordance with Shannon’s diversity (*H*) and species richness(*S*); it was measured to be18 > 38 > 5 > 0 d. There were significant differences in the pattern and level of carbon sources used by the phytoplankton-associated bacteria. In addition, the principle component analyses (PCA) suggested that the first principle component (PC1) and the second principle component (PC2) explained 46.76% and 21.49% of the total variation for bacterial community, respectively. Redundancy analysis (RDA) revealed that cell abundance of phytoplankton was negatively correlated with the *AWCD*_590nm_, amino acids and other functional indexes. Therefore, the data suggest that there are differences in the phytoplankton-associated bacterial community functional diversity during different static stages of water samples collected from the drinking water reservoir.

## 1. Introduction

Phytoplankton and bacteria play important roles in maintaining the structure and function of aquatic ecosystems [1,2]. In the past few decades, the relationships between phytoplankton and bacteria were widely evaluated, including mutualism, parasitism and competition [3,4,5]. In the mutualistic relationships, heterotrophic bacteria utilize the excretion from phytoplankton as carbon sources [6,7,8]. Different phytoplankton community compositions lead to differences in kinds of organic carbon which the microbial community can utilize for their growth [9]. Compared with the massive literatures focused on succession of phytoplankton and bacteria community composition in different water bodies [10,11], the functional diversity of associated bacteria during degradation of the phytoplankton from drinking water reservoir is not well determined, although bacteria species have vital services involvement in organic carbon decomposition in this freshwater ecosystem [12].

The community-level physiological profiles technique called BIOLOG was successfully used to determine the bacterial community metabolic fingerprints in a drinking water reservoir [13]. BIOLOG ECO micro-plate has thirty one different sole carbon sources, including amino acids, phenolic compounds, carbohydrates, polymers, carboxylic acids and amines [14]. These carbon sources can be utilized by phytoplankton-associated bacteria, and the metabolic fingerprints can be employed to reveal the functional diversity of bacterial community [14]. Zhu [15] used 454 pyrosequencing and the BIOLOG technique to explore the dynamic changes of the bacterial communities during cyanobacterial blooms, and suggested that the bacterial communities associated to the four cyanobacterial genera had a very high richness, and the bacterial communities varied in both functional and structural diversities [15]. However, the literature focused on the metabolic characteristics of the bacterial community associated with the phytoplankton in drinking water reservoirs is limited.

A drinking water reservoir is important for the safety of urban water supply [16]. In the last few years, phytoplankton and bacteria are increasingly attracting researchers’ attention because the changes of phytoplankton and bacteria can reduce the drinking water quality [13,17]. Ma [18] demonstrated that the phytoplankton community was mainly made of diatoms in spring, and blue-green algae became dominant in summer, in a water source reservoir. In addition, Zhang [13] found the spatial dynamics of bacterial community structure in the same reservoir. Meanwhile, the interaction between functional diversity of microbial communities and dynamics of algal species can drive the nutrients transformation and water quality changes in drinking water reservoirs. Unfortunately, no information is available about the functional diversity of associated bacteria during degradation of the phytoplankton in a water source reservoir.

Extending our knowledge of the carbon metabolic characteristics of the phytoplankton-associated bacteria in the reservoir will improve our understanding of the links between phytoplankton and microbial communities. Therefore, the main aim of present work is to determine the associated bacterial community metabolic functional diversity during the degradation of phytoplankton. To this end, the specific aims of this work are: (i) To examine the community composition and cell abundance of phytoplankton during degradation stages; and (ii) to determine the metabolic profiles of associated bacterial communities over the degradation of phytoplankton.

## 2. Experimental Section

### 2.1. Sampling Description

The water samples were collected from JIN PEN reservoir (N34°02′45″, E108°12′04″). The height of the dam reaches 130 m, area of 4.55 × 10^3^ m^2^, with a water volume of 2 × 10^8^ m^3^, and it serves as a drinking water supply sources for Xi’an and near cities [18]. Near-surface water samples were collected by sterilized vertical organic glass hydrophore and placed into the ethylene plastic bucket (10 L). The hydrophore and bucket were sterilized by ethanol disinfection in the lab and rinsed with sterile distilled water for three times before sampling. After sampling, the water samples were transported immediately to the Key Laboratory of Shaanxi Environmental Engineering, School of Environmental and Municipal Engineering, Xi’an University of Architecture and Technology (SEME, XAUAT, Xi’an, China) within 24 h. In the laboratory, the samples were kept in darkness; the phytoplankton can be biodegraded naturally without external interference [19]. The phytoplankton community composition and functional diversity of the associated bacteria were determined after 0, 5, 18 and 38 d, respectively.

### 2.2. Water Quality Determination

Depth of water (Dep), dissolved oxygen (DO), pH, water temperature (T),oxidation reduction potential (ORP), conductivity (Con), turbidity and chlorophyll *a* were examined using a multi-probe water quality sonde (Hydrolab DS5, HACH, USA) in situ when sampling. Total nitrogen (TN) and total phosphorus (TP) of the water sample were determined using spectrophotometer (UV-mini 1240, Japan), according to the method described by Ma et al. [18].

### 2.3. Phytoplankton Community Composition Analysis

According to the method described by Qiu [20], 1% Lugol’s solution was added to the water samples to fix the phytoplankton before counting. Amicroscope (OLYMPUS CX31, Japan) was used to observe cell abundance and community composition of phytoplankton, identified according to the freshwater algae of China [21].

### 2.4. Associated Bacterial Community Metabolic Determination

To determine the functional metabolic fingerprints of the phytoplankton-associated bacterial community, the BIOLOG method was employed to investigate the utilization patterns of carbon source [15]. BIOLOG ECO is a plate of 96 wells, which has3 parallels, contains one blank control and thirty-one different carbon substrates, including amines, phenolic compounds, carbohydrates, carboxylic acids, polymers and amino acids (Table 1) [22,23].

According to the method used by Zhu with little modifications [15], briefly, onaclean bench, water samples were filtrated through a 1.2 µm pore size membrane (Isopore Membrane Filters, Millipore). Then, 150 μL of filtrate was added into each well of the ECO micro-plate using an electronic pipette [15]. The inoculated ECO micro-plate was then incubated at 28 ± 2 °C in a dark chamber (Jinghong, Shanghai, China) for 240 h [23,24]. The absorbance at 590 nm was determined using an Elisa reader (BIOLOG Company, Hayward, CA, USA). Bacterial community activity in ECO micro-plate was expressed as average well color development (*AWCD*), and was expressed as the following formula:

*AWCD* = ∑(*C_i_* − *R*)/31 (1)

where *R* was the value of blank control, *C_i_* was the value of carbon substrates well, and negative optical density (OD_590nm_) values were set to zero, *C_i_* − *R* ≥ 0 [14].

Indexes of bacterial community functional diversity were showed as species richness (*S*), Simpson’s diversity (*D*) and Shannon’s diversity (*H*).

*S* was the number of utilized carbon sources well in the ECO plate, and *AWCD* > 0.2 represents that the carbon substrate has been utilized [25]:

*S* = ∑(*C_i_* − *R*) (*C_i_* − *R* > 0.2) (2)

*H* and *D* were calculated as the equations below [14,25]:

*H* = −Σ *P_i_* × ln*P_i_* (3)

*D* = 1 − Σ *P_i_*^2^ (4)

where *P_i_* = (*C_i_–R*)/ Σ(*C_i_–R*).

In this work, the data of 120 h incubation [13] was used for *AWCD*_590nm_, community diversity indexes, carbon substrate utilization, principle component analysis (PCA) and redundancy analysis (RDA).

### 2.5. Redundancy Analysis (RDA) of Phytoplankton Community Composition and Associated Bacterial Community Metabolic Fingerprints

To investigate whether attributes of the phytoplankton accounted for significant changes in the functional diversity of the associated bacterial community, RDA was employed to reveal the bacterial metabolic fingerprints with phytoplankton community characteristics as explanatory variables. The following parameters were used as descriptors of the phytoplankton community: Shannon’s diversity index (*H_p_*); total number of phytoplankton cells L^−1^ (*N*); and the relative abundance of *Melosira* (*M*). Shannon’s diversity index for the phytoplankton community (*H_p_*) was calculated as described by Rooney-Varga [11]. The data of 120 h of *AWCD*, *H*, *S*, *D* and the six kinds of carbon substrates were taken for response variables, which represented the associated bacterial community metabolic fingerprints.

### 2.6. Statistical Analysis

Data statistical analysis was carried out using the Sigma Plot (Version 16.0) software package for windows. A parametric one-way analysis of variance (ANOVA) test followed by Tukey–Kramer HSD tests was used. Principle component analysis (PCA) was performed using SPSS version 18.0 software for windows (SPSS Inc., Chicago, IL, USA), and the first two PC1 and PC2 were selected. CANOCO for Windows (Version 4.5) was used to conduct redundancy analysis (RDA) ordinations.

## 3. Results and Discussion

### 3.1. Water Quality

The results of the water quality parameters monitored are as shown in Table 2. Temperature is an important factor affecting growth of the phytoplankton [26,27]. Though the environmental temperature already reached 25 °C, the water temperature was only 12 °C. The nitrogen to phosphorus (N:P) ratio of the water sample was 43.According to Redfield’s law, when the ratio of total nitrogen to total phosphorus exceeds 16: 1, phosphorus is considered to be the limiting factor [20]. The concentration of *Chl-a*, TN and TP were 1.54 µg/L, 0.98 mg/L and 0.012 mg/L, respectively. Based on the eutrophication evaluation criteria for Chinese lakes and reservoirs [18], the water sample was considered to be moderately eutrophicated.

### 3.2. Phytoplankton Cell Concentration and Composition

**Figure 1:**
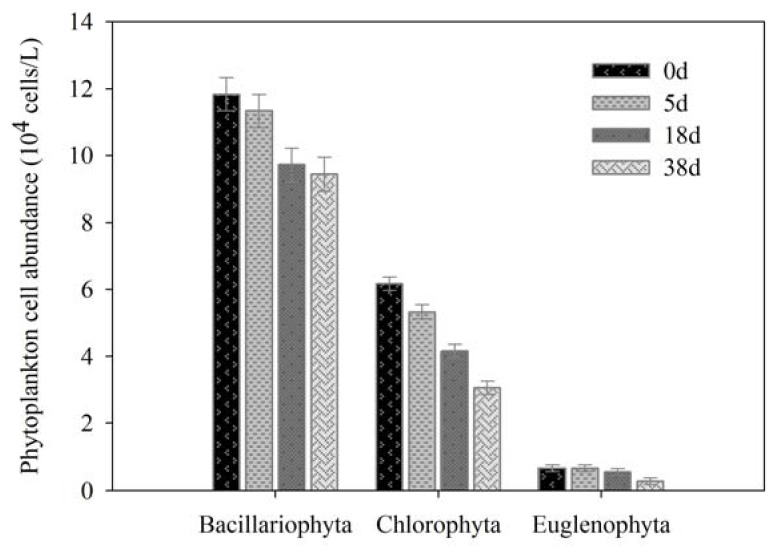
Variations of phytoplankton cell abundance (phylum).

**Figure 2:**
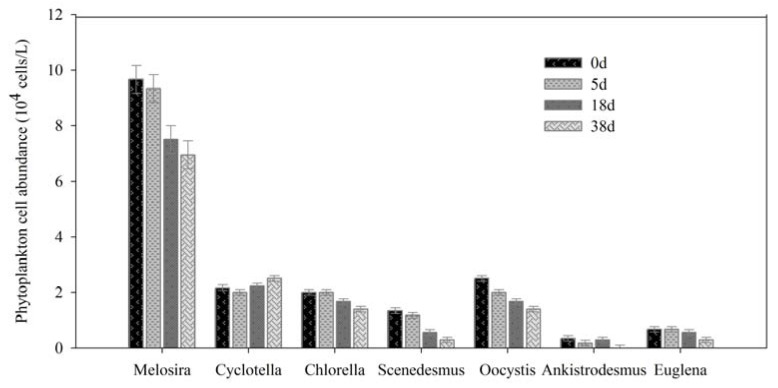
Variations of phytoplankton cell abundance (genera).

Phytoplankton play an important role in maintaining the stability of aquatic ecosystems as primary producers, where by abundance and community composition can directly influence and indicate the water quality [28,29]. A total of three phyla and seven genera or species of phytoplankton were identified, with *Bacillariophyta* having the largest number of species, reaching to 11.33 × 10^4^ cells /L at the beginning, and *Melosira* was dominant species in JINPEN reservoir in spring (Figure 1 and Figure 2).

The degradation rate of *Euglena* was fastest, and decreased 58.33% from 0 to 38 d, while the smallest degradation of *Bacillariophyta* was 20.19% (Figure 1). In addition, *Ankistrodsemus* was degraded, but *Cyclotella* increased slightly (Figure 2). It was suggested that *Ankistrodsemus* decomposed easier, whereas *Cyclotella* had great vitality and was hard to degrade. It may be connected to their physiological characteristics.

As shown in Table 3, the Shannon’s diversity (*H_p_*) of phytoplankton decreased from 0 d (1.479) to 5 d (1.432), and increased slightly in 18 d, then reduced in 38 d with 1. The relative abundance of *Melosira* (*M*) was ordered as: 38 d (0.543) > 5 d (0.538) > 18 d (0.519) > 0 d (0.518) (Table 3).

### 3.3. Bacterial Community Metabolic Profiles

**Figure 3:**
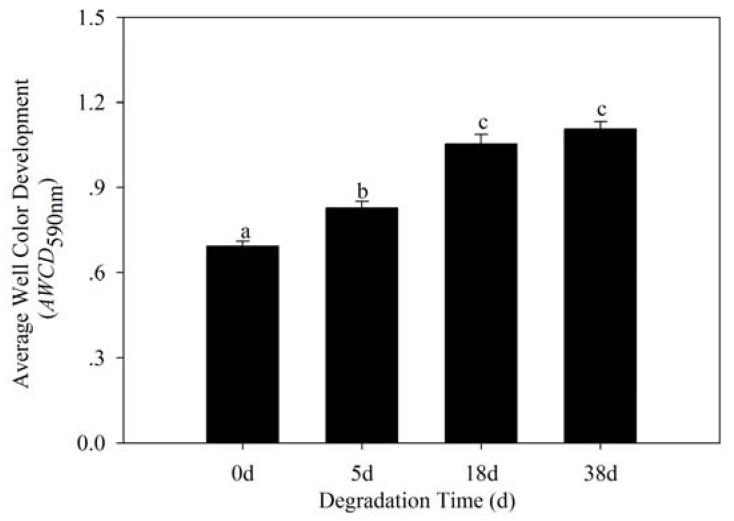
Average well color development (*AWCD*_590nm_) of the phytoplankton-associated bacterial community. Data are expressed as the mean values ± SD (*n* = 3). Different letters above the bars indicate significant differences (*p* < 0.05) assessed by Tukey–Kramer HSD.

The associated bacterial community metabolic activity (*AWCD*_590nm_) significantly improved with degradation of phytoplankton (Figure 3).

As shown in Figure 3, the *AWCD*_590nm_ increased during the degradation periods from 0 to 38 d. The highest *AWCD*_590nm_ (1.107) was found in 38 d, and the lowest in 0 d at 0.694.

One-way analysis of variance showed that *AWCD*_590nm_ of degraded periods was significantly higher than the beginning (0 d); however, *AWCD*_590nm_ of 18 and 38 d had no significant differences (*p* < 0.05). These results revealed that the ability to utilize carbon substrates for the associated bacterial community increased with degradation of phytoplankton, indicating that metabolic activity of the bacterial community enhanced.

The significant highest species richness (*S*) was found in 18 d, and the lowest was in 0 d (*F* = 12.62, *p* < 0.05). The Shannon’s diversity (*H*) in 18 d was 3.297 ± 0.007, which was significantly higher than that of 0 d with 3.130 ± 0.037 (*F* = 19.71, *p* < 0.05) (Table 4). The Simpson’s index (*D*) was in accordance with Shannon’s diversity (*H*) and species richness(*S*), it was measured that 18 > 38 > 5 > 0 d. However, there were no significant differences for those three diversity indexes among 5, 18 and 38 d (Table 4). The most important reason for this phenomenon is that most organic carbon was released from the cell of algae, and more carbon sources were used by the bacterial community during 18 d.

As shown in Table 5, the utilization of amino acids, amines, polymers, phenolic compounds, carbohydrates and carboxylic acids by the phytoplankton-associated bacteria were different with different degradation time. Carbohydrates were most utilized by the associated bacteria at 38 d, and amino acids were most utilized by the associated bacteria at 18 d, respectively. Carboxylic acids were most metabolized at 5 d (Table 5).

**Figure 4:**
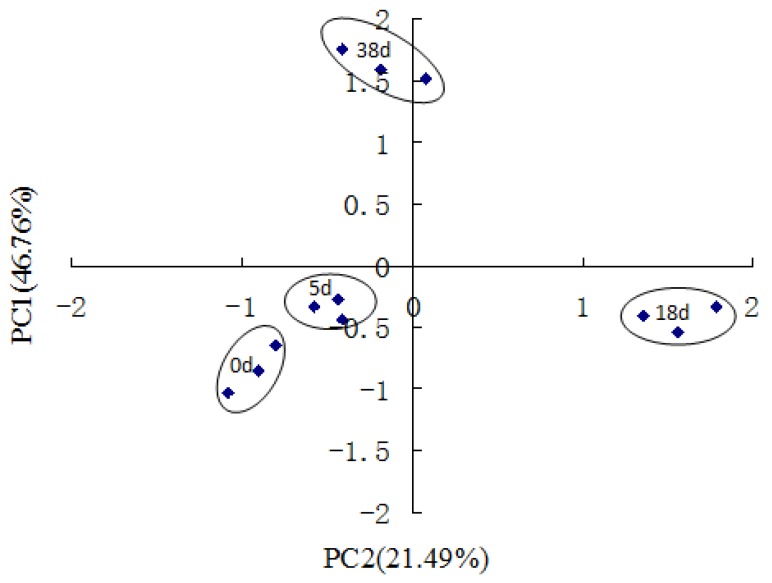
Principal components analysisof thirty one different sole carbon substrate metabolic profiles of the associated bacteria during degradation of phytoplankton. PC1 explained 46.76% of the total variance and PC2 explained 21.49%, respectively.

**Figure 5:**
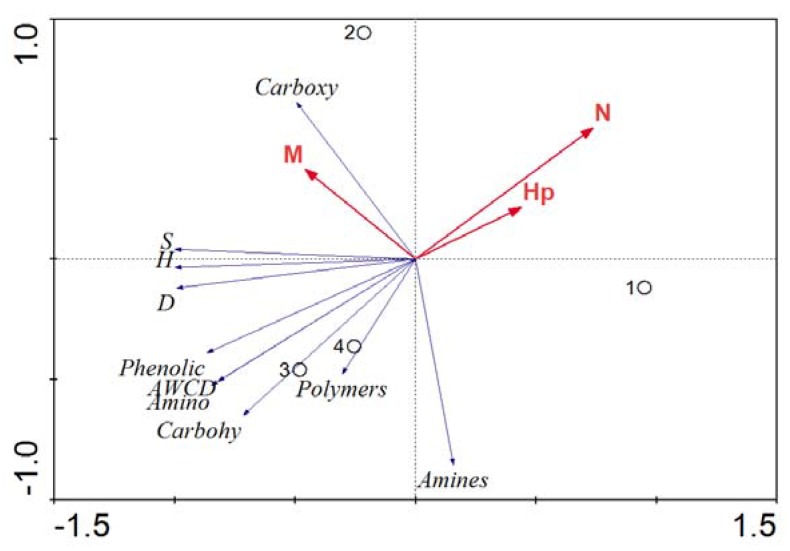
Redundancy analysis (RDA) ordination tri-plots of the phytoplankton community and the associated bacterial community metabolic fingerprints. *N* represents the cell abundance of phytoplankton, *M* represents the relative abundance of *Melosira*, *Hp* represents Shannon–Wiener index of phytoplankton. *AWCD* is average well color development. *H*, *S* and *D* are the associated bacterial community diversity indexes. Amino, Amines, Polymers, Phenolic, Carbohy and Carboxy represent amino acids, amines, polymers, phenolic compounds, carbohydrates and carboxylic acids, respectively. 1, 2, 3 and 4 represent 0, 5, 18 and 38 d, respectively.

Principal components analysis (PCA) suggested that the significant bacterial community functional metabolic profile discrimination existed among different degradation times (Figure 4). As shown in Figure 5, the first two principles explained 68.25% of the total variance. PC1 and PC2 explained 46.76% and 21.49% of the variance, respectively. It was suggested that the 18 and 38 d associated bacterial community functional diversity was more unstable than that of the 5 d water sample (Figure 4). Therefore, these data revealed that the metabolic fingerprints of the associated bacterial community changed with degradation of phytoplankton. The higher discrimination of carbon substrates in the principle component analysis of the data of carbon source utilization is shown in Table 6, including D,L-a-glycerol, 2-hydroxy benzoic, y-hydroxybutyric acid, L-threonine and glycyl-L-glutamic acid.

### 3.4. Correlation between the Phytoplankton Community and Associated Bacterial Community Metabolic Fingerprints (RDA)

Figure 5 shows the influence of phytoplankton community variables on the functional diversity of phytoplankton-associated bacteria. The first axis of the ordination explained 95.1% of the total variance, while the second axis, an additional 3.3%. The first ordination axis had the highest eigenvalue (0.951); therefore, the changes along the first ordination axis of phytoplankton community will have the greatest impact on the functional diversity of the associated bacteria. *N* was negatively correlated with the *AWCD*, amino acids and other functional indexes. *M* was positively correlated with the carboxylic acids, *S* and *H*. From the intersample distances, it can be seen that the associated bacterial community functional diversity of 18 and 38 d was similar (Figure 5).

Several works have shown the interactions between phytoplankton and bacterial communities, and the dynamics of phytoplankton community composition have been observed to correlate with changes in bacterial community composition [11,30,31]. Rooney-Varga [11] investigated the relationship between phytoplankton and bacterial community dynamics in ocean environment conditions, and suggested that species composition shifts in the attached bacteria and phytoplankton communities were correlated. There is also evidence that differences in the quality of organic carbon, produced by different types of phytoplankton, cause changes in the structure composition of bacterial communities utilizing this organic carbon [32]. Therefore, changes in phytoplankton community composition may influence the composition of bacterial communities that function as part of the microbial loop. However, the effect of the changes in phytoplankton community composition on bacterial metabolic functional diversity was not well understood.

In the present work, we determined whether relationships between bacteria and phytoplankton community dynamics existed. It had been suggested that the *AWCD* values increased with degradation of phytoplankton, and bacterial metabolic activities increased, indicating that carbon sources released by phytoplankton increased. This result is consistent with a study conducted by Sun et al. [33], who determined release of colloidal and particulate nutrition in the course of decomposition of cyanobacteria, which showed colloidal organic carbon reached five times that of the beginning. Tranvik [34] also examined the effects of organic matter on the growth of bacteria in lake water, and suggested that colloidal organic matter served as a supplementary source of nutrition that could promote the growth of bacteria and protists. Whereas, the associated bacterial diversity indexes (*H*, *S* and *D*) increased from 0 to 18 d, and decreased at 38 d. The reason may be that the organic matter released by phytoplankton differed with different degradation days. Sun et al. [33] revealed that the content of organic carbon increased firstly and then decreased in the course of decomposition of cyanobacteria. Dickerson et al. [1] used the BIOLOG method to determine spatial and temporal community-level physiological profiles for three fresh water lakes of different trophic levels, and found bacterial communities utilized the carbon guilds similarly between sites within the three lakes. When the metabolic profile of each lake was compared, Lake Bradford and Moore Lake were more similar to one another than to Lake Munson, the eutrophic lake. The reason most important was that, with increased nutrient loading, bacterial activities increase, while bacterial diversity decrease.

Sarmento et al. [35] used micro autoradiography to quantify the preferences of the heterotrophic prokaryote on dissolved organic carbon derived from phytoplankton species, and found that the vast range of different types of organic molecules available in the sea selects and maintains the high levels of diversity described for marine bacterioplankton. In this study, we found that there was a significantly different carbon source utilization pattern in associated bacterial community. PCA revealed differences in associated bacterial community functional diversity among different stages; it showed that the associated bacterial community changed significantly throughout the degradation time. In order to explain the relationships between the phytoplankton and bacterial community further, we used RDA with phytoplankton community characteristics as explanatory variables (Figure 5). RDA results indicated an association between phytoplankton and associated bacterial community functional diversity that would be expected if specific bacteria–phytoplankton interactions occurred. Amin et al. [36] teased apart a bacterial consortium associated with a globally-distributed diatom, and found that a *Sulfitobacter* species promotes diatom cell division via secretion of the hormone indole-3-acetic acid, synthesized by the bacterium using both diatom-secreted and endogenous tryptophan. Dang et al. [37] found marine bacteria were known to be colonizers of particulate matter and were likely to be utilizing organic compounds provided by phytoplankton cells [11]. Hold et al. [38] observed that different bacterial assemblages were associated with different din flagellate species, suggesting species-specific interactions. Liu et al. [2] used quantitative PCR and 454 pyrosequencing methods to investigate bacterial communities composition affected by phytoplankton community succession in a drinking water reservoir, and found that the distinct succession of phytoplankton community could mediate the temporal dynamics of the bacterial community in the Tingxi Reservoir. Some advanced molecular techniques, such as stable isotope probing (SIP), have been used to detect the interaction between bacteria and algae [39,40] and identify the bacteria responsible for contaminant biodegradation [41,42,43]. Denitrifying bacterial communities (such as *nirS*-type), explored by using Illumina sequencing, will also be used to identify the nitrogen-cycling metabolic microbe [44]. Meanwhile, SIP and ecological network analysis [45] could be used to detect the associated bacteria during degradation of phytoplankton in a drinking water reservoir in future.

## 4. Conclusions

Although many studies have shown the interactions between phytoplankton and bacterial communities, little is known about how these communities interact at the species composition and functional levels in drinking water reservoir ecosystems. In this work, light microscopy and BIOLOG techniques were used to reveal the correlation between phytoplankton and associated bacterial communities, and the metabolic fingerprints of associated bacteria related to the degradation of phytoplankton were determined. The results suggested that *AWCD* values of associated bacteria increased with degradation time of phytoplankton. While the associated bacterial community diversity index increased in the early stages and then decreased, the highest bacterial diversity index was observed in 18 d. More organic carbon released from the cell of algae can be utilized by bacterial community in 18 d. Principal components analysis revealed a significant difference in the associated bacterial community functional metabolic profiles among different static times of phytoplankton. Redundancy analysis indicated that the total number of cells of phytoplankton was negatively correlated with the metabolic function of the associated bacteria. The results from this work suggested that decomposed phytoplankton could influence the metabolic activity of associated bacteria in a drinking water reservoir.

## Figures and Tables

**Table 1:** Sole carbon substrates in BIOLOG used in the present work [22,23].

Carbohydrates	Carboxylic Acids	Amino Acids	Polymers	Phenolic Compounds	Amines
D,L-a-Glycerol a-D-lactose β-Methyl-D-glucoside I-Erythritol D-Cellobiose D-Mannitol D-Xylose Glucose-1-phosphate N-Acetyl-D-glucosamine D-Galactonic acid y-lactone	Pyruvic acid Methyl ester y-Hydroxy butyric acid D-Galacturonic acid a-Ketobutyric acid D-Glucosaminic acid D-Malic acid Itaconic acid	L-Arginine L-Threonine L-Serine L-Phenylalanine L-Asparagine Glycyl-L-glutamic acid	a-Cyclodextrin Glycogen Tween40 Tween80	4-Hydroxy benzoic acid 2-Hydroxy benzoic acid	Phenylethyl-amine Putrescine

**Table 2:** Water quality of the sampling sites.

Dep (m)	T (°C)	DO (mg/L)	pH	ORP (mV)	Con (µS/cm)	Tur (NTU)	*Chl-a* (µg/L)	TN (mg/L)	TP (mg/L)
0.5	12.06	9.91	7.98	347	174	9	1.54	0.98	0.012

**Table 3:** Relative abundance of *Melosira* (*M*) and Shannon’s diversity (*H_p_*) of phytoplankton.

Degradation Time	Shannon’s Diversity (*H_p_*)	Relative Abundance of *Melosira* (*M*)
0 d	1.479	0.518
5 d	1.432	0.538
18 d	1.453	0.519
38 d	1.299	0.543

**Table 4:** Species richness (*S*), Simpson’s diversity (*D*) and Shannon’s diversity (*H*) indexes of phytoplankton-associated bacterial community.

Degradation Time	Species Richness (*S*)	Simpson’s Diversity (*D*)	Shannon’s Diversity (*H*)
0 d	23.667 ± 1.155b	0.949 ± 0.002b	3.130 ± 0.037b
5 d	27.667 ± 0.577a	0.957 ± 0.001a	3.257 ± 0.015a
18 d	28.333 ± 0.577a	0.960 ± 0.0004a	3.297 ± 0.007a
38 d	27.667 ± 1.528a	0.958 ± 0.002a	3.261 ± 0.040a

Note: Data are expressed as the mean values ± SD (*n* = 3). The same capital letter after the data represents no significant difference by Tukey–Kramer HSD (*p* < 0.05).

**Table 5:** Variance analysis of utilization of the six groups of carbon sources (amino acids, amines, polymers, phenolic compounds, carbohydrates and carboxylic acids) located in the BIOLOG ECO plate by the associated bacterial community.

Degradation Time	Amino Acids	Amines	Polymers	Phenolic Compounds	Carbohydrates	Carboxylic Acids
0 d	0.62 ± 0.07b	0.74 ± 0.25a	0.95 ± 0.20a	0.30 ± 0.09a	0.59 ± 0.07b	0.86 ± 0.02b
5 d	0.84 ± 0.04b	0.39 ± 0.09a	0.93 ± 0.05a	0.78 ± 0.24b	0.65 ± 0.02b	1.16 ± 0.05a
18 d	1.36 ± 0.12a	0.67 ± 0.06a	1.00 ± 0.07a	1.62 ± 0.06c	0.96 ± 0.06a	0.90 ± 0.03b
38 d	1.18 ± 0.15a	0.92 ± 0.45a	1.38 ± 0.12b	1.00 ± 0.04b	1.02 ± 0.08a	1.09 ± 0.06a

Note: The data represent the means and standard errors (*n* = 3). The same capital letter after the data represents no significant difference by Tukey–Kramer HSD (*p* < 0.05).

**Table 6:** Higher discrimination of carbon substrates in the principle component analysis of the data of carbon sources utilization by the associated bacterial community.

Carbon Substrates Located in BIOLOG ECO Plate	PC1 Score	PC2 Score
Tween80	−0.703	0.521
Glycogen	0.768	−0.006
D-Cellobiose	0.880	0.342
ß-Methyl-D-Glucoside	0.806	0.428
D-Mannitol	−0.868	0.437
N-Acetyl-D-Glucosamine	0.862	0.072
D-Glucosaminic Acid	−0.848	0.425
D,L-a-Glycerol	0.939	−0.222
2-Hydroxy Benzoic	0.964	−0.029
y-Hydroxybutyric Acid	0.949	−0.255
L-Phenylalanine	0.743	0.579
L-Threonine	0.945	0.048
Glycyl-L-Glutamic Acid	0.938	−0.285
